# Right Meralgia Paresthetica After a Laparoscopic ProGrip™ Bilateral Implant for Inguinal Hernia Repair: A Case Report

**DOI:** 10.7759/cureus.105059

**Published:** 2026-03-11

**Authors:** Mauro Geller, Mendel Suchmacher, Rafael F Coelho

**Affiliations:** 1 Clinical Immunology, Instituto de Pós-Graduação Médica Carlos Chagas, Rio de Janeiro, BRA; 2 Urology, Instituto do Câncer do Estado de São Paulo/Faculdade de Medicina Universidade de São Paulo (ICESP/FMUSP), Sao Paulo, BRA

**Keywords:** extrinsic neural compression, lateral femoral cutaneous nerve, meralgia paresthetica, neuropathic pain, self-fixating mesh implant

## Abstract

Meralgia paresthetica is a neuropathic condition generally secondary to extrinsic compression on the lateral femoral cutaneous nerve. Intense local pain is the chief presenting symptom, diagnosis is based on clinical grounds, and the disease is responsive to the removal of the causal agent and antineuralgic medication. We report the case of a 67-year-old male patient who had a ProGrip™ (Medtronic, Minneapolis, MN, USA) (a self-fixating mesh) implanted through laparoscopy after a robotic bilateral inguinal hernia repair. The next day, the patient complained of numbness in the anterolateral aspect of the right thigh along with intolerable stabbing pain. Meralgia paresthetica was diagnosed based on a positive Tinel sign, followed by symptomatic relief after corticosteroids + lidocaine injection. The manifestation was attributed to ProGrip™, and the device was removed. Pain was controlled after discharge with oral pregabalin plus cyclobenzaprine. In a retrospective analysis, no classical risk factors of meralgia paresthetica (e.g., obesity, tight belts, or former local procedures) were identified for the patient under discussion. The authors could not find any similar case in medical literature.

## Introduction

Meralgia paresthetica is a neuropathic pain syndrome generally due to the entrapment or compression of the lateral femoral cutaneous nerve (LFCN) [1]. Pregnancy, diabetes mellitus, and obesity represent risk factors. Age at diagnosis ranges from the fifth to the sixth decade of life (women are prone to a higher age range). Some authors claim there is a male general predominance [2,3]. The anatomical basis for meralgia paresthetica is analogous to that of carpal tunnel syndrome: the portion of the anterior lamina of the iliac fascia located between the ileopubic tract and the inguinal ligament - as well as the ileopubic tract itself - are tough fibrotic tissue [4]. LFCN runs through this "aponeuroticofascial tunnel," which begins at the ileopubic tract and ends at the inguinal ligament. Extrinsic compression on this rigid tunnel triggers the local inflammation and corresponding clinical manifestations.

Meralgia paresthetica presents diverse etiologies. Nevertheless, direct trauma, extrinsic compression near or at the inguinal ligament, and idiopathic meralgia paresthetica are the most common mechanisms. Meralgia paresthetica presenting symptoms are (1) skin hypoesthesia, dysesthesia, pain, paresthesia, or numbness, (2) normal lower limb strength and reflexes, and (3) back pain (if proximal lesions are present) [1,5]. Dermal symptoms can be referred to the anterolateral region or the upper half of the lateral surface of the thigh, if the anterior or posterior branches of LFCN, respectively, are involved [2]. Both a positive Tinel sign (pain on percussion at LFCN emergence from under the inguinal ligament) and a positive pelvic compression test (pain relief after applying a constant downward force on the pelvis while the patient is lying laterally) can be found. Meralgia paresthetica’s main differential diagnosis is L2-L3 radiculopathy. Semiotics associated with this condition, which allow differentiation, are the following: (1) symptoms are referred outside the distribution area of the LFCN, and (2) there is crural muscle wasting with a diminished knee jerk [3,6].

Meralgia paresthetica diagnosis is based on clinical presentation and a therapeutic trial with a local nerve block (see below) [7]. Nevertheless, if warranted, the most sensitive imaging techniques to document entrapment or compression of LFCN are crural neurography (peripheral nerve magnetic resonance imaging) and peripheral nerve ultrasound (US) (≥5 mm enlargement of LFCN’s cross-sectional area, nerve fascicle edema expressed as hypoechogenicity, or extrinsic bodies around LFCN) [4,7]. Management of the etiological agent is pivotal for meralgia paresthetica’s full recovery. Specific therapeutic modalities for meralgia paresthetica are (1) pharmacological management (amitriptyline, pregabalin) [1,8]; (2) US-guided nerve block of LFCN using local anesthetics (bupivacaine, lidocaine) and/or corticosteroids (dexamethasone) (both diagnostic and therapeutic) [1,7]; (3) neurolysis (incision of the inguinal ligament, the fascia lata, or the internal oblique abdominal muscle to decompress LFCN) [1,2,8]; and (4) neurectomy (surgical transection the LFCN at the inguinal ligament) [3,9].

ProGrip™ laparoscopic self-fixating mesh

ProGrip™ laparoscopic self-fixating mesh (Medtronic, Minneapolis, MN, USA) is a device used for inguinal hernia repair procedures. It is structured with more than 5,000 atraumatic microgrips and is devoid of tacks, a characteristic associated with a lower degree of post-surgical pain. Even though more than 40% of its mass is bodily absorbed over time, it seems that in certain individuals, as it was in our case report, the ProGrip™ uptake rate did not provide timely protection from meralgia paresthetica [10]. Acute urinary retention is one reported post-surgical complication, with no recurrences or major complications reported [11].

## Case presentation

**Figure 1:**
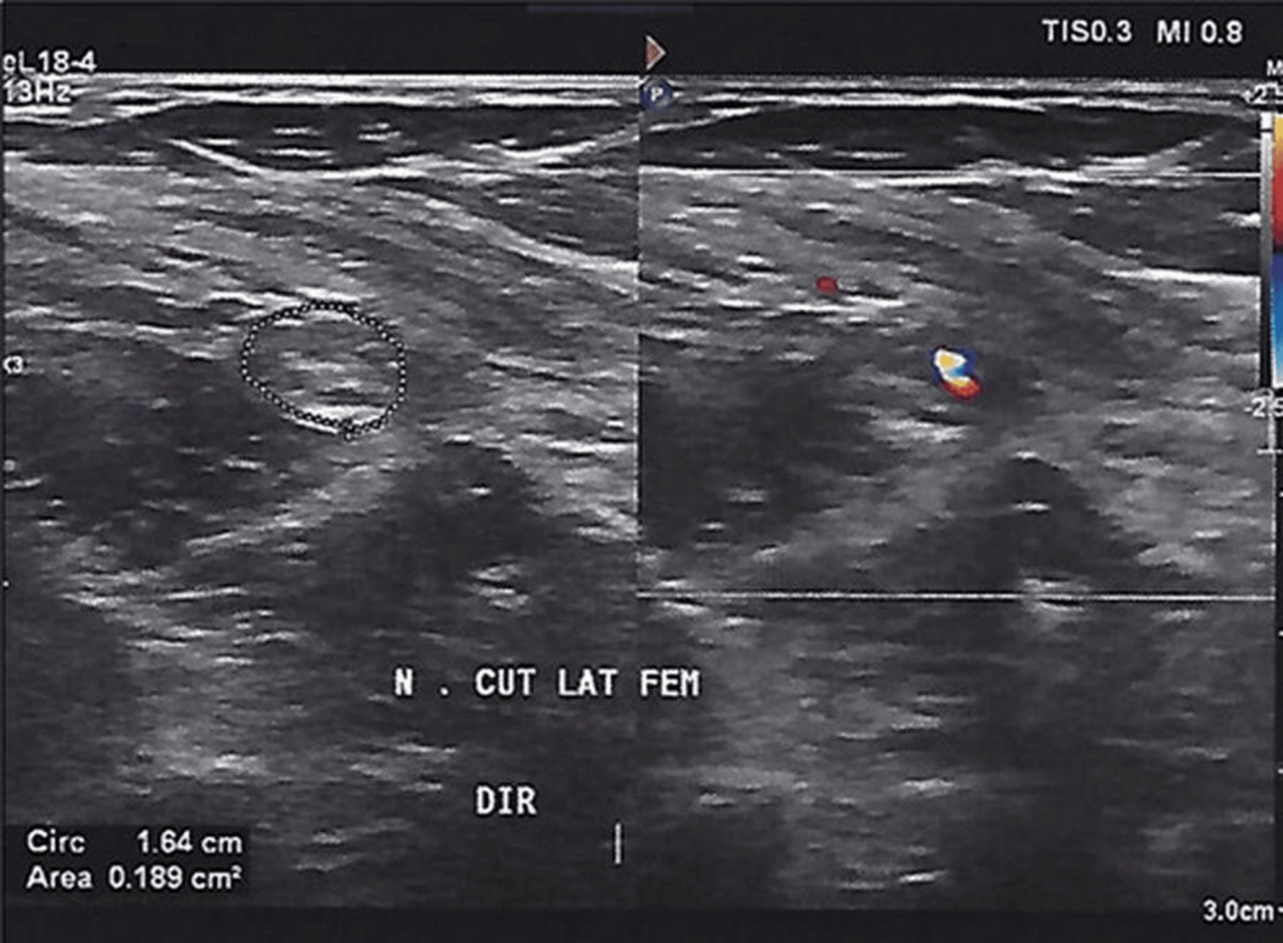
Right LFCN presented as a hypoechogenic and thick fusiform image (highlighted), scanned above the inguinal ligament. Focal loss of fascicular pattern is also observed. Doppler US showed peri- and intraneural vascularization increase (reference values for cross-sectional area: 0.9-1.5 cm2). LFCN: lateral femoral cutaneous nerve; US: ultrasound

A 67-year-old male patient, with a normal BMI, underwent a robotic prostatectomy due to benign prostatic hyperplasia, as well as robotic bilateral hernia repair through a totally extraperitoneal repair technique, followed by ProGrip™ laparoscopic self-fixating mesh bilateral placement. His past history was negative for spine diseases, peripheral neuropathies, or surgical procedures in the inguinal region. He denied wearing unfit clothing, such as tight pants or belts. The procedure was uneventful. Biopsy yielded nodular stromal and glandular prostatic hyperplasia, a mild lymphocytic infiltrate, and no atypical cells. The next morning, the patient complained of numbness in the anterolateral aspect of the right thigh and intolerable stabbing pain when in the orthostatic position. Meralgia paresthetica was diagnosed based on a positive Tinel sign, followed by symptomatic relief after US-guided local injection of betamethasone sodium phosphate and betamethasone dipropionate, 2.0 and 5 mg, respectively, +lidocaine 1% (the patient did not allow any other semiotics maneuvers since pain was unbearable during body mobilization). Oral pregabalin and IV ketoprofen, as 300 mg daily and 100 mg twice daily regimens, respectively, were prescribed. The condition was attributed to extrinsic compression by the ProGrip™, and the device was removed through laparoscopy. Neuropathic pain disappeared the next day, but numbness persisted (the marked pain improvement was partially attributed to lidocaine + betamethasone injection, previously injected). The patient received three weekly intramuscular injections of betamethasone sodium phosphate and betamethasone dipropionate, 2.0 and 5 mg, respectively, after discharge, as physicians took into consideration the inflammatory character of the primary lesion and the possibility of neuropathic pain relapse. A peripheral nerve US was performed one week after hospital discharge (Figure 1).

LFCN neuritis was the radiological diagnosis. Numbness was referred by the patient as mild at the date of the exam. The patient was still on pregabalin plus cyclobenzaprine.

## Discussion

Assuming the position of the self-fixating mesh, i.e., adjacent to the right inguinal ligament/ileopubic tract, and negative history for preexisting or current back pain complaints, the authors hypothesized that the neuropathic pain mechanism was due to mechanical compression of the inferior edge of this device on the "aponeuroticofascial tunnel" [5,6]. The latter phenomenon could have occurred on the anterior branch of the LFCN, since the patient located the pain on the anterolateral aspect of the thigh (correspondingly, anatomical variations are present in one quarter of individuals diagnosed with meralgia paresthetica) [12]. The pain was described by the patient as excruciating, different from the depictions in the literature where the symptom is detailed qualitatively rather than quantitatively, and the intensity of this level is seldom referred to [7,8]. Temporal relationship with ProGrip™ placement, anatomical proximity of the device to LFCN, and the clinical picture consistent with meralgia paresthetica pointed to local nerve block followed by ProGrip™ extraction surgery as the best possible conduct.

As in most instances, diagnosis in this case was clinical, corroborated by pain relief after local nerve block with corticosteroids + lidocaine and sustained pain resolution after removal of the suspected causative mesh [4]. We could not find imaging data on US Doppler performed over LFCN lesions associated with meralgia paresthetica in the context of post-ProGrip™ placement in the literature [4,7], as was described in this case (Figure 1). This could be an additional original aspect in this case report. Fortunately, in this case, the compression on LFCN did not last long enough for long-term complications to develop, such as permanent local hyposensitivity.

## Conclusions

The authors could not find other clinical reports on meralgia paresthetica caused by mechanical LFCN compression exerted by a self-fixating mesh installed for laparoscopic hernia repair in the medical literature. Clinical presentation was typical, with continuous numbness and intense pain complaint triggered by orthostatic position over the LFCN anatomical distribution. Diagnosis was made based on clinical grounds and on a diagnostic/therapeutic trial (local corticosteroid and lidocaine injection). Symptomatic response was obtained after device removal and anti-inflammatory prescription, followed by slow additional improvement.
